# Simulated trials: in silico approach adds depth and nuance to the RCT gold-standard

**DOI:** 10.1038/s41746-021-00492-7

**Published:** 2021-08-11

**Authors:** Leia Wedlund, Joseph Kvedar

**Affiliations:** 1 Harvard Medical School, Boston, MA USA; 2 Mass General Brigham, Boston, MA USA

**Keywords:** Clinical trial design, Research data

For decades, Randomized Controlled Trials (RCTs) have reigned as the gold-standard in evidence-based medicine, but these studies are expensive, time-intensive, and often take place under very artificial treatment conditions that are not replicated in real-world clinics once the intervention is approved^1^. Often under scrutiny for underrepresentation of women and minorities, RCTs also tend to study a younger, healthier population than the true target market, at times making it difficult to generalize the results^2,3^.

Despite these limitations, RCTs remain our closest approximation of causality. However, clinical trial simulations add depth and nuance to the data gathered in RCTs, validating or challenging RCT findings in a more generalizable setting. Clinical trial simulations use real-world data sets to run multiple trial protocols (scenarios) on medical records databases. Simulations are frequently used to design optimal RCT protocols, but they can also provide opportunities for further study after the RCT concludes, allowing researchers to study a larger sample of real-world patients or adjust patient inclusion criteria to test a new hypothesis.

A recent simulation by Chen et al. using the OneFlorida medical records database, illustrates how in silico simulations crafted using a large and diverse patient dataset can provide additional nuance when compared with the results of an RCT^4^. The authors set out to re-create the safety profile of donepezil 10 mg as seen in a phase III randomized controlled trial using deidentified patient records of people prescribed donepezil, and after running their simulation on two very demographically distinct groups of patient records, they ultimately demonstrated that the demographic make-up of the patient population may affect the apparent safety profile of donepezil.

The original phase III double-blinded donepezil trial population was 73.5% White, and patients in the control arm of the RCT (taking 10 mg donepezil) had an 8.3% rate of Serious Adverse Events (SAEs)^5^. Using medical records from the OneFlorida database, applying the same inclusion criteria as the original study, and selecting for a patient population with the *same racial make-up as the original RCT*, the simulation authors set out to determine the safety profile of donepezil 10 mg using real-world data. The simulation results were strikingly similar to the original phase III trial: the simulation showed an 8.9% rate of SAEs amongst real-world patients taking donepezil 10 mg (compared to 8.3% in the phase III RCT). At face value, this is encouraging evidence that the original RCT safety profile results are replicable and accurate, but it would be a waste to stop there.

The power of a simulation is that researchers can run nearly infinite scenarios using real-world data at little to no cost. Simply re-creating the original RCT safety results using proportional sampling to obtain the same type of nondiverse patient population included in the phase III trial does not address the issues of RCT underrepresentation discussed above. Thus, it is important to consider how the simulation results vary when more diverse patient populations are included. In that vein, the authors ran the same simulation again using a much more diverse patient population, which was randomly sampled from the OneFlorida database and more representative of Florida’s demographic breakdown.

In this second, more diverse simulation population, only 28% of the patient records included belonged to White patients, and the results were strikingly dissimilar from the original RCT (which was 73.5% White). This second run of the simulation, using a more diverse population, showed an SAE rate of 15.5% (vs 8.3% in the original RCT). This is not the first evidence that donepezil may affect some patient populations differently: a 2015 prospective study of ethnically diverse patients taking donepezil for Alzheimer’s Disease found that the drug provided no statistically significant benefit, while a comparable study populated with white non-Latino Alzheimer’s patients found a modest benefit^6^. The powerful modifying effect of demographics on SAE rates in this simulation provides us with a more nuanced understanding of donepezil’s safety profile. While race may not be the only modifier at play here (we cannot rule out the possibility of hidden confounders such as treatment center), the dramatic effect of demographics in this simulation suggests that the original RCT may not have captured a higher SAE rate amongst minorities, making it difficult to generalize the safety results to all populations.

RCTs remain the gold-standard of medicine, but as digital models improve, we can finally address cost, diversity, and size constraints to create an even stronger standard for the future. Simulations may never fully replace RCTs, but as the size and complexity of our medical records data grows, simulations provide an opportunity to replace the static conclusions of a traditional clinical trial with a more nuanced and dynamic understanding of medicine that better serves all stakeholders.

## References

[1] Morrison K (2001). Randomised controlled trials for evidence-based education: some problems in judging’what works’. Eval. Res. Educ..

[2] Hoel AW (2009). Under-representation of women and ethnic minorities in vascular surgery randomized controlled trials. J. Vasc. Surg..

[3] Heiat A, Gross CP, Krumholz HM (2002). Representation of the elderly, women, and minorities in heart failure clinical trials. Arch. Intern. Med..

[4] Chen Z (2021). Exploring the feasibility of using real-world data from a large clinical data research network to simulate clinical trials of Alzheimeras disease. NPJ Digital Med..

[5] Doody RS (2012). Efficacy and safety of donepezil 23 mg versus donepezil 10 mg for moderate-to-severe Alzheimer’s disease: a subgroup analysis in patients already taking or not taking concomitant memantine. Dementia Geriatric Cognit. Disord..

[6] Tinklenberg JR (2015). Donepezil treatment in ethnically diverse patients with Alzheimer disease. Am. J. Geriatric Psychiatry.

